# Direct endothelial junction restoration results in significant tumor vascular normalization and metastasis inhibition in mice

**DOI:** 10.18632/oncotarget.1942

**Published:** 2014-05-02

**Authors:** Vijayendra Agrawal, Sony Maharjan, Kyeojin Kim, Nam-Jung Kim, Jimin Son, Keunho Lee, Hyun-Jung Choi, Seung-Sik Rho, Sunjoo Ahn, Moo-Ho Won, Sang-Jun Ha, Gou Young Koh, Young-Myeong Kim, Young-Ger Suh, Young-Guen Kwon

**Affiliations:** ^1^ Department of Biochemistry, College of Life Science and Biotechnology, Yonsei University, Seoul 120-749, Republic of Korea,; ^2^ College of Pharmacy, Seoul National University, 599 Gwanak-ro, Gwanak-gu, Seoul 151-742, Republic of Korea,; ^3^ Department of Pharmacy, College of Pharmacy, Kyung Hee University, Seoul 130-701, Republic of Korea,; ^4^ Korea Research Institute of Chemical Technology (KRICT), 141 Gajeong-ro, Yuseong-gu, Daejeon 305-343, Republic of Korea,; ^5^ Department of Neurobiology, School of Medicine, Kangwon National University, Chuncheon 200-701, Republic of Korea,; ^6^ National Research Laboratory of Vascular Biology and Stem Cells, Korea Advanced Institute of Science and Technology (KAIST), Daejeon 305-701, Republic of Korea,; ^7^ Vascular System Research Center, Kangwon National University, Kangwon-Do, Republic of Korea

**Keywords:** Vascular permeability, Hypoxia, Normalization, Epithelial-to-mesenchymal transition, Sac-1004

## Abstract

Tumor blood vessels are leaky and immature, which causes inadequate blood supply to tumor tissues resulting in hypoxic microenvironment and promotes metastasis. Here we have explored tumor vessel modulating activity of Sac-1004, a recently developed molecule in our lab, which directly potentiates VE-cadherin-mediated endothelial cell junction. Sac-1004 could enhance vascular junction integrity in tumor vessels and thereby inhibit vascular leakage and enhance vascular perfusion. Improved perfusion enabled Sac-1004 to have synergistic anti-tumor effect on cisplatin-mediated apoptosis of tumor cells. Interestingly, characteristics of normalized blood vessels namely reduced hypoxia, improved pericyte coverage and decreased basement membrane thickness were readily observed in tumors treated with Sac-1004. Remarkably, Sac-1004 was also able to inhibit lung and lymph node metastasis in MMTV and B16BL6 tumor models. This was in correlation with a reduction in epithelial-to-mesenchymal transition of tumor cells with considerable diminution in expression of related transcription factors. Moreover, cancer stem cell population dropped substantially in Sac-1004 treated tumor tissues. Taken together, our results showed that direct restoration of vascular junction could be a significant strategy to induce normalization of tumor blood vessels and reduce metastasis.

## INTRODUCTION

Tumor angiogenesis features leaky, dilated, saccular, and tortuous blood vessels; the extent and type of abnormality varies with tumor type and location [1-3]. Tumors commonly exhibit an abnormally thick basement membrane and perivascular cells with abnormal morphology [3]. Leaky blood vessels result in hemorrhage that increases interstitial fluid pressure and limits vascular perfusion, leading to a hypoxic and acidotic microenvironment [1, 3, 4]. In this microenvironment, cancer, endothelial, and stromal cells secrete various growth factors (e.g., vascular endothelial growth factor [VEGF], angiopoietin [Ang]2, platelet derived growth factor (PDGF), placental growth factor, transforming growth factor (TGF)-alpha, and hepatocyte growth factor), establishing an endless loop of non-productive angiogenesis [2, 3]. In addition, hypoxia and hypoxia inducible factor (HIF)-1 triggers a series of events known as epithelial-to-mesenchymal transition (EMT), which not only promotes primary growth but also metastasis of tumor [5, 6].

Previous studies showed that tumor therapies targeting angiogenic growth factors are much effective when given in combination with cytotoxic (chemo and radio) agents [1]. It was suggested that anti-angiogenic therapy, to some extent, corrects the flaw in structure and function of tumor blood vessels. This normalization of vessels decreases leakiness and improves perfusion resulting in enhanced delivery and efficacy of cytotoxic agents [7-9]. Though highly effective, the efficiency of combination therapy is restrained by the limited duration of normalization window [8]. Other studies reported that increased expression of Ang1 was able to stabilize blood vessels [10-12]. Similarly, inhibition of Ang2 was shown to improve endothelial cell-cell junction, induce tumor vessel normalization [13], and inhibit metastasis [14]. Various other investigators have extensively studied the tumor vascular normalization phenomenon [4, 15], and the importance of vascular junction in tumor microenvironment modulation is irrefutable. Nevertheless, the effects of direct potentiation of endothelial junction on tumor vascular normalization and metastasis has not been clearly demonstrated.

Recently we developed a vascular-leakage-blocking molecule Sac-1004, which promisingly reduced VEGF-induced endothelial permeability in cell-based assays [16, 17]. Sac-1004 was found to reorganize actin cytoskeleton at endothelial cell-cell junction to form cortical actin ring via cAMP/Rac/Cortactin pathway. This cortical actin ring enhanced cell-cell junction integrity by upregulation of junction proteins such as vascular endothelial (VE)-cadherin. Interestingly, in addition to VEGF, Sac-1004 could dampen other agonists (thrombin and histamine)-induced vascular hyperpermeability too via similar mechanism [18].

Here, we found that Sac-1004 could substantially inhibit vascular leakage in pathological conditions like diabetic retina and tumor. This provided an effective way to study the correlation between vascular permeability and tumor behavior. Remarkably, significant vascular normalization was observed in Sac-1004 treated tumors. Additionally, reduced hypoxia together with increased vascular stability seemingly reduced the extent of metastasis in treated tumor models. This coincided with down-regulation of various EMT-regulating transcription factors, along with a drop in cancer stem cell (CSC) population, in Sac-1004 treated tumors. Altogether, our study demonstrates that direct inhibition of vascular leakage by molecules like Sac-1004 could provide a novel strategy to induce vessel normalization and inhibit metastasis.

## RESULTS

### Sac-1004 is capable of decreasing tumor vascular leakage

Since vascular hyperpermeability is a long recognized hallmark of tumors [19], we evaluated the effect of Sac-1004 on vascular leakage *in vivo*. VEGF is one of the prominent molecules responsible for leakiness of tumor vessels [20-22]; thus we initially investigated the effect of Sac-1004 in retinas of VEGF-treated and diabetic mice [23, 24]. Sac-1004 significantly attenuated VEGF-induced retinal vascular leakage (Supplementary Figure S2A,B). Likewise, retinal hyperpermeability in streptozotocin-induced diabetic mice was inhibited by intravitreous/intravenous injections of Sac-1004 (Supplementary Figure S2C-G). Since Sac-1004 was shown to stabilize junction protein VE-cadherin *in vitro* [18], we analyzed junction protein levels in diabetic mice retina. Sac-1004 treatment restored the expressions of VE-cadherin and occludin in diabetic retinas (Supplementary Figure S2H,I).

**Figure 1:**
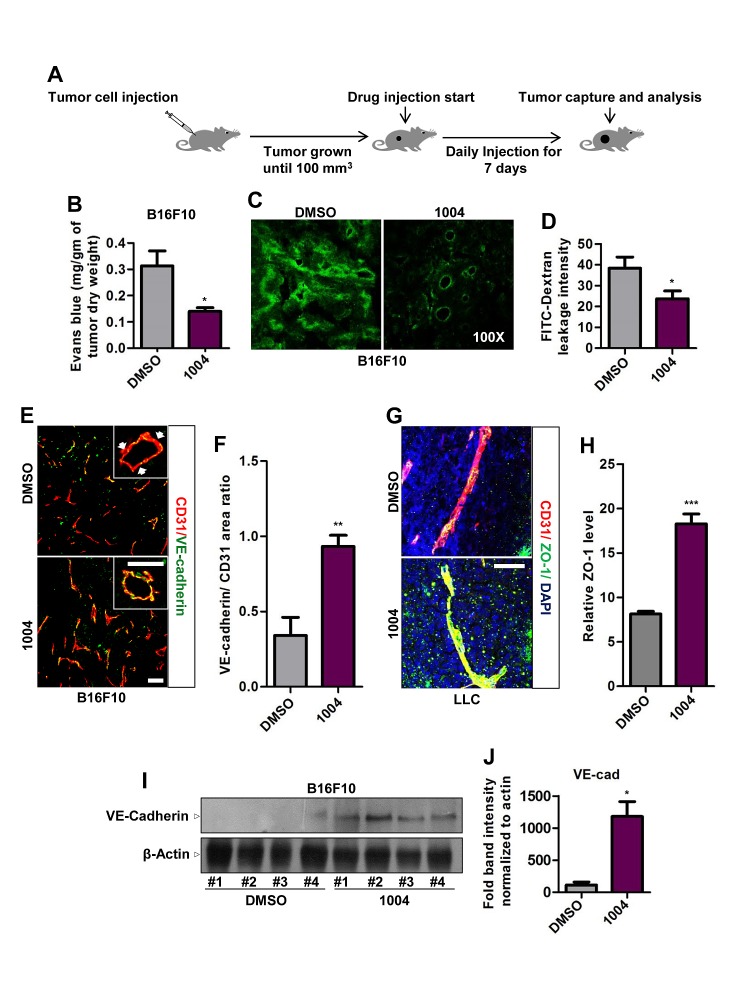
Sac-1004 reduces vascular leakage with concomitant increase in junction integrity in tumor blood vessels (A) Schematic plan for the administration of Sac-1004 (indicated as 1004) or control (DMSO) to tumor-bearing mice. (B) B16F10 tumor-bearing mice (n = 5) were injected with Sac-1004 or control as in (A) and tumor vascular leakage was quantified by the Evans blue method. (C) Vascular leakage was assessed by FITC-dextran. (D) Iimages shown in (C) were quantified using ImageJ software. Three sections per tumor (100 μm apart) (n = 5) were photographed and quantified. (E) Immunofluorescence staining of B16F10 tumor sections, treated with Sac-1004 or control, for CD31 and VE-cadherin. Arrows indicate discontinuity in VE-cadherin staining. Scale bar, 100 μm (50 μm in inset). (F) Quantification of immunofluorescence images shown in (E) using Multi Gauge software (n = 5). (G) LLC tumor sections, treated with Sac-1004 or control were costained for CD31, ZO-1 and DAPI. Scale bar, 50 μm. (H) Images shown in (G) were quantified using ImageJ software (n = 5). (I) Western blot analysis of B16F10 tumors treated with Sac-1004 or control for VE-cadherin. (J) VE-cadherin and actin blots from (I) were quantified using ImageJ software. *P<0.05; **P<0.01; ***P<0.001 (Student's *t*-test). Data are represented as mean ± s.e.m.

Next we treated B16F10 and LLC tumors, grown subcutaneously in mice, with Sac-1004 and examined leakage using Evans blue dye and FITC-dextran (Figure 1A). We found that Sac-1004 treatment significantly reduced tumor vessel leakiness (Figure 1B-D and Supplementary Figure S4B,C); however, tumor growth was not affected in either tumor (Supplementary Figure S3B,S4A). Interestingly, significantly increased number of VE-cadherin-positive vessels were observed in B16F10 tumors receiving Sac-1004 (Figure 1E,F). Additionally, VE-cadherin lining was discontinuous in the control group but continuous in the treatment groups (Figure 1E). Sac-1004-treated LLC tumors also exhibited similar continuous lining of VE-cadherin and ZO-1 (Supplementary Figure S4D and Figure 1G). Increase in VE-cadherin level was confirmed by Western blot of tumor proteins (Figure 1I,J). Angiopoietins are well known regulators of vascular stability [25] and Ang2 serves as a promoter of vascular junction destabilization [14, 26]. Immunostaining of B16F10 tumor sections showed substantially reduced Ang2 expression in Sac-1004 group (Supplementary Figure S3H,I). Also reduction in Ang2 expression in Sac-1004-treated HUVECs was observed in time-dependent manner (Supplementary Figure S4H,I).

Sac-1004, by itself, did not exert any cytotoxic effects on various tumor cells *in vitro* (Supplementary Figure S3A), nor did it have significant effect on EC proliferation, apoptosis, and necrosis (Supplementary Figure S3C-E). These results suggest that Sac-1004 can block tumor vascular leakage without affecting tumor growth and deterioration of tumor endothelial cells.

### Sac-1004-mediated Leakage Inhibition Enhances Tumor Vascular Perfusion and Alleviates Hypoxia

**Figure 2:**
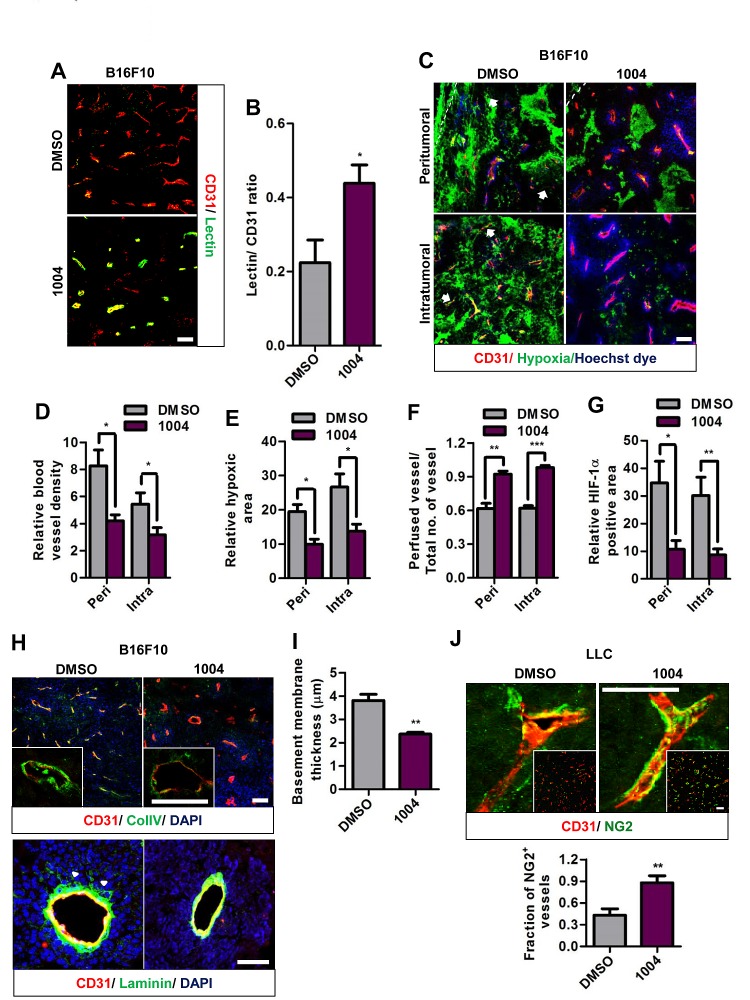
Sac-1004 improves vascular perfusion, alleviates hypoxia and normalizes tumor blood vessels in tumors (A) Immunofluorescence staining of B16F10 tumor sections (n = 5), treated with Sac-1004 or control, for CD31 and tomato lectin. Scale bar, 100 μm. (B) Images shown in (A) were quantified using ImageJ software. (C) Immunohistochemical analysis of B16F10 tumor sections (n = 5) for CD31, hypoxia, and vascular perfusion (Hoechst dye) in the peritumoral and intratumoral zone. Arrows indicate non-perfused vessels. Scale bar, 100 μm. (D-F) Quantification of immunofluorescence images shown in (C) with Multi Gauge software. (G) Quantification of HIF-1α positive area using Multi Gauge software. (H) B16F10 tumor sections (n = 5), treated with Sac-1004 or control, were stained for CD31 and ColIV (up)/ laminin (bottom). Scale bar, 100 μm (50 μm in insets). Arrowheads indicate the point of detachment between basement membrane and endothelial cells. Scale bar, 50 μm. (I) Quantification of basement membrane thickness in B16F10 tumor vessels shown in (H) using Multi Gauge software. (J) Immunofluorescence staining of LLC tumor sections (n = 5) for CD31 and NG2. Scale bar, 50 μm (100 μm in insets). Quantification was done using Multi Gauge software. *P<0.05; **P<0.01; ***P<0.001 (Student's *t*-test). Data are represented as mean ± s.e.m.

Interstitial hypertension caused by vessel leakiness collapses blood vessels and impedes vessel perfusion. We found that Sac-1004 treatment increased the ratio of perfused blood vessels as shown by Hoechst staining (Figure 2C,F and Supplementary Figure S5A) and by tomato lectin (Figure 2A,B and Supplementary Figure S4F,G).

Also, Sac-1004 treatment significantly reduced hypoxia in both tumors (Figure 2C,E and Supplementary Figure S5A,B). Consistently, expression of HIF-1α was found to be decreased in B16F10 tumors treated with Sac-1004 (Figure 2G and Supplementary Figure S3G). Interestingly, we observed that vascular density in peri- and intratumoral regions of B16F10 tumor was decreased by Sac-1004 treatment (Figure 2C,D); however, such a change was not evident in LLC tumors (Supplementary Figure S5A). The intrinsic characteristics and response to therapeutic agents may differ among tumor types. Overall, these findings indicate that Sac-1004-induced vascular-permeability-reduction increases patency and results in diminution of hypoxic tumor microenvironment.

### Reduction of Vascular Leakage Normalizes Tumor Blood Vessels

We further evaluated B16F10 and LLC tumor sections for basement membrane thickness and pericyte coverage; signatures of normalized tumor vessels [4]. Both the tumor vessels exhibited abnormally thickened basement membranes when stained for Collagen IV. Upon treatment with Sac-1004, this abnormal thickness was significantly alleviated (Figure 2H (up),I and Supplementary Figure S5D,E). Immunostaining of B16F10 tumor sections for laminin also revealed detached basement membrane structures, which was prevented by Sac-1004 treatment (Figure 2H (down)); however, LLC tumor sections didn't exhibit any significant difference (Supplementary Figure S5C).

Staining of LLC tumor sections for pericyte marker NG2 and desmin revealed pericyte coverage deficiency, as previously noted [27]. However, Sac-1004-treated tumors exhibited marked increase in extent and fraction of NG2 and desmin-positive pericyte covered vessels (Figure 2J and Supplementary Figure S5F,G). Unlike LLC tumors, B16F10 tumor blood vessels are covered with pericytes as noted previously [28] and Sac-1004 treatment did not induce any further change (Supplementary Figure S5H).

Extravasation of plasma components via leaky vessels creates an inflammatory environment, recruiting macrophages. Tumor-associated macrophages (TAMs) release several growth factors which further escalate tumor growth [2] and is also associated with poor prognosis in cancer therapy [29, 30]. Examination of B16F10 tumor showed that Sac-1004 treatment significantly reduced F4/80^+^ TAM population (Supplementary Figure S5I,J). Altogether, above results indicate that Sac-1004-mediated leakage reduction could alter abnormal tumor vessels to normalized forms.

### Vascular-Leakage-Inhibition Enhances Chemosensitivity of Tumor

**Figure 3:**
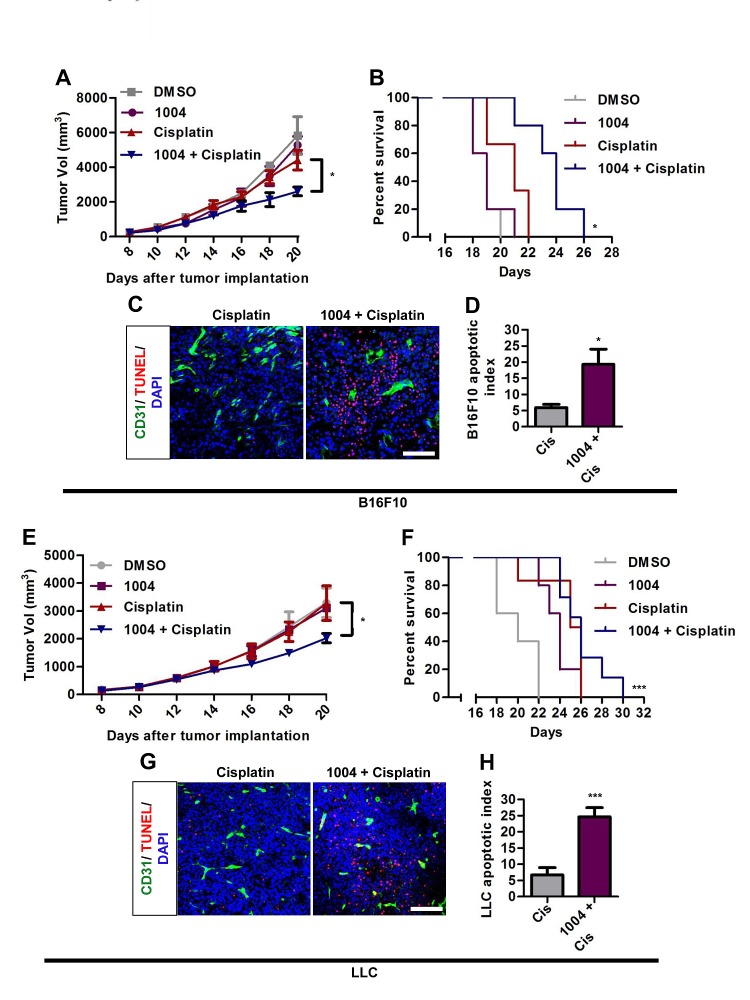
Sac-1004 augments the tumor-growth suppressing effect of cisplatin in tumor-bearing mice B16F10 and LLC tumor-bearing mice were intravenously injected with Sac-1004 alone or in combination with cisplatin, and tumor volumes (A and E) were measured on alternate days for 2 weeks (n = 8 tumors per group from two independent experiments). Also survival of mice was monitored (B and F). Each point in Kaplan-Meier curve represents the percent of surviving mice. Mice with tumor size greater than 5000 mm^3^ were considered dead. The *p* values were calculated using log rank test (n = 8 mice per group). Immunofluorescence analysis of Sac-1004 and/or cisplatin-injected B16F10 (C) and LLC (G) tumors (two dose of Sac-1004 and a single dose of cisplatin as combination therapy) for TUNEL-positive cells (n = 5). Scale bar, 100 μm. (D) Quantification of apoptotic tumor cells as shown in (C) using ImageJ software. (H) Quantification of apoptotic tumor cells as shown in (G) using ImageJ software. *P<0.05; ***P<0.001 (Student's *t*-test except survival curve data). Data are represented as mean ± s.e.m.

Reducing vascular leakage increases the efficacy of drug delivery to tumor [1]. Combination therapy of B16F10 tumor-bearing mice with Sac-1004 and cisplatin showed that tumor growth was not much affected by cisplatin alone; however, the growth inhibition was significantly augmented in combination group (Figure 3A). Consequently, survival of tumor-bearing mice was substantially increased in the combination group (Figure 3B). Similar patterns were found in LLC tumors treated with Sac-1004 and cisplatin (Figure 3E,F). Consistently, TUNEL-positive apoptotic cells were markedly increased in B16F10 and LLC tumors of combination treatment group (Figure 3C,D,G,H). These results suggest that Sac-1004 could enhance the access of cytotoxic drugs to tumor cells and thereby improve therapeutic response.

### Sac-1004 Mediates Reduction of Lung Metastasis in MMTV-PyMT Mice

**Figure 4:**
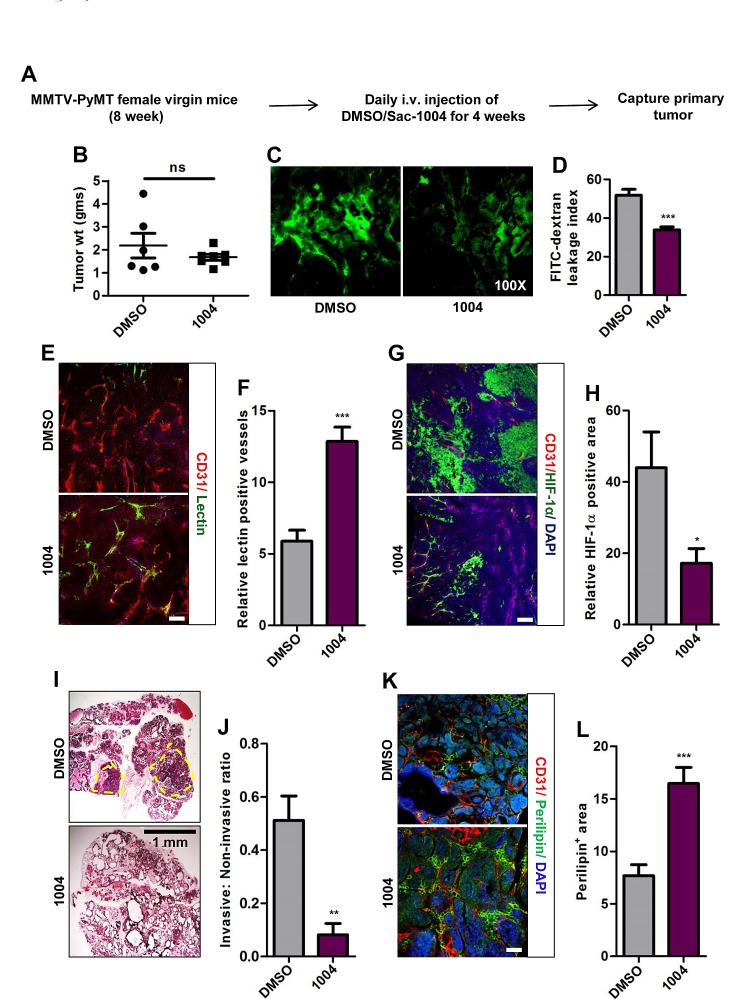
Sac-1004 reduces vascular leakage, HIF-1α expression and malignancy of breast tumor in MMTV-PyMT mice model (A) Schematic plan for the treatment of MMTV-PyMT female mice with Sac-1004. (B) Comparison of breast tumor weight of MMTV mice (n = 7) after treatment with Sac-1004. ns, not significant. (C) MMTV mice were injected with FITC-dextran before capture of tumor (n = 7). (D) FITC-dextran leakage from blood vessels as shown in (C) was quantified using ImageJ software. (E) Immunostaining of MMTV tumor sections (n = 7) for CD31 and lectin. (F) Quantitation of lectin positive vessels as shown in (E) using ImageJ software. (G) MMTV tumor sections were stained for CD31 and HIF-1α (n = 7 mice). (H) Quantitation of HIF-1α positive tumor area as shown in (G) using ImageJ software. (I) Hematoxylin and eosin staining of MMTV breast tumor sections (n = 7). Yellow circle denotes invasive zone. Scale bar, 1 mm. (J) Quantitation of ratio of invasive and non-invasive regions from figure shown in (I) using Multi Gauge software. (K) Immunohistochemical staining of MMTV tumor sections with CD31 and perilipin (n = 7 mice). (L) Quantitation of perilipin positive area from Figure shown in (K) using ImageJ software. Scale bar, 100 μm (in all images). *P<0.05; **P<0.01; ***P<0.001 (Student's *t*-test). Data are represented as mean ± s.e.m.

We also investigated the effect of vascular-leakage inhibition on spontaneously growing breast tumor as in MMTV-PyMT mice (Figure 4A). MMTV mice receiving Sac-1004 displayed reduction in tumor-vascular-leakage in coherence with B16F10 and LLC tumors (Figure 4C,D). Though no significant change was observed in tumor weight of control and drug-treated mice tumors (Figure 4B), Sac-1004 treatment did increase vascular perfusion (Figure 4E,F) and reduced HIF-1α expression (Figure 4G,H). Interestingly, Sac-1004 caused reduction in invasive tumor fraction in breast tumors (Figure 4I,J). This was confirmed by an increase in the expression of perilipin-1, a marker for fat cells (Figure 4K,L).

**Figure 5:**
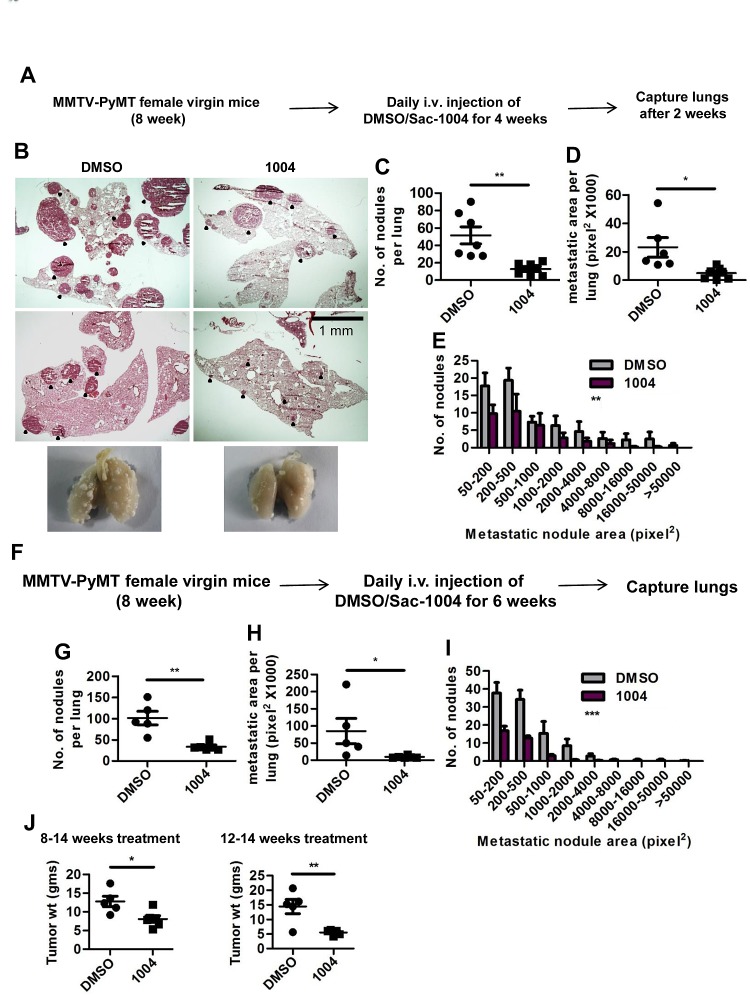
Sac-1004 reduces the extent of metastasis in MMTV-PyMT mice (A) Schematic plan for the administration of Sac-1004 to MMTV mice for metastatic study. (B) Hematoxylin and eosin staining of lung sections from MMTV mice (n = 7). Black dots indicate metastatic nodules. Scale bar, 1 mm. (C-E) Quantitation of the number of nodules (C), metastatic area (D), and distribution of metastatic nodules (E) per lung based on the staining shown in (B) using Multi Gauge software. (F) Schematic plan for the long-term treatment of MMTV mice with Sac-1004. (G-I) Quantitation of the number of nodules (G), metastatic area (H), and distribution of metastatic nodules (I) per lung from mice receiving long-term treatment using Multi Gauge software (n = 7). (J) Comparison of breast tumor weight from mice receiving long-term treatment (8-14 weeks) or late treatment (12-14 weeks) of Sac-1004 (n = 7). *P<0.05; **P<0.01; ***P<0.001 (Student's *t*-test except for metastatic nodule distribution, which was analyzed by 2 way ANOVA). Data are represented as mean ± s.e.m.

In concordance with B16F10 and LLC tumors, MMTV tumors also exhibited increased VE-cadherin and reduced Ang2 expression in blood vessels upon Sac-1004 treatment (Supplementary Figure S6A-D). Intensive pericyte coverage, thinned basement membrane, and reduced macrophage population were evident in Sac-1004-treated MMTV tumor sections (Supplementary Figure S6E-I). Noticeably, Sac-1004 treated MMTV mice showed significant reduction in number, size, and distribution of lung-metastatic colonies (Figure 5B-E). Numerous, big-sized metastatic nodules were observed in control lungs; however, fewer and small nodules were present in treated ones (Figure 5B). Mice receiving long-term treatment (8-14 weeks) also displayed similar reduction in lung metastasis (Figure 5F-I). Surprisingly, the weight of breast tumors was also reduced in mice receiving long-term or late (12-14 week) treatment of Sac-1004 (Figure 5J); however, no change in tumor cell apoptosis was observed. Thus, Sac-1004 was able not only to normalize the blood vessels in spontaneously growing tumor, but also to prevent metastasis to a great extent.

### Sac-1004 Mediates Decrease in Lung and Lymph Node Metastasis in B16BL6 Mice

**Figure 6:**
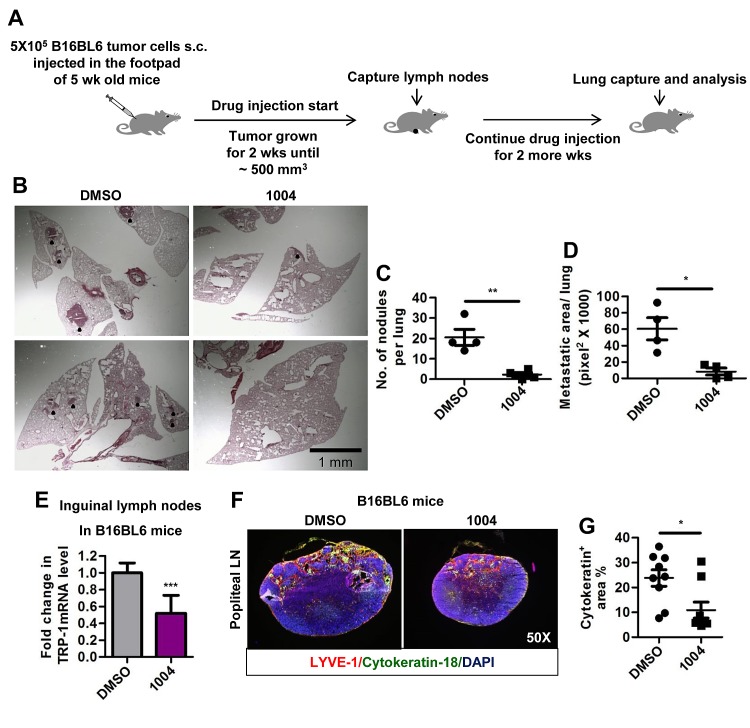
Sac-1004 reduces lung and lymph node metastasis in B16BL6 foot-pad metastasis mice model (A) Schematic plan for the administration of Sac-1004 and capture of lymph nodes and lungs from B16BL6 mice model. (B) Hematoxylin and eosin staining of lung sections (n = 5 mice). Black dots indicate metastatic nodules. Scale bar, 1 mm. (C and D) Quantitation of the number of nodules (C) and metastatic area (D) per lung from the sections stained in (B) using Multi Gauge software. (E) RNA samples from inguinal lymph nodes (n = 10) were used to quantitate the expression of TRP-1 using real-time PCR. (F) Sections of popliteal lymph nodes (n = 10) were immunostained for LYVE-1 and cytokeratin-18 (melanocyte marker). (G) Quantitation of cytokeratin positive area from the sections stained in (F) using Multi Gauge software. *P<0.05; **P<0.01; ***P<0.001 (Student's *t*-test). Data are represented as mean ± s.e.m.

Another widely used model for spontaneous lung and lymph node metastasis involves injecting B16BL6 cells into the right footpad of mice, from where they metastasize to lymph nodes and lungs [31]. In consistence with MMTV tumor metastasis, B16BL6 tumor-bearing mice receiving Sac-1004 (Figure 6A), exhibited reduced number and size of lung metastatic colonies (Figure 6B-D). In addition, there was a sharp drop in cytokeratin-18 positive cells migrating to popliteal lymph nodes (Figure 6F,G). Real-time-PCR analysis of inguinal lymph nodes for tyrosinase-related-protein-1 (melanocyte marker) also confirmed decreased lymph node metastasis in Sac-1004 treated mice (Figure 6E); however, lymphatic vessel number or structure seemed unaffected (Supplementary Figure S6J). Hence, Sac-1004 was able to reduce distant and regional metastasis in B16BL6 mice model.

### Sac-1004 Reduces EMT in Tumor Cells

**Figure 7:**
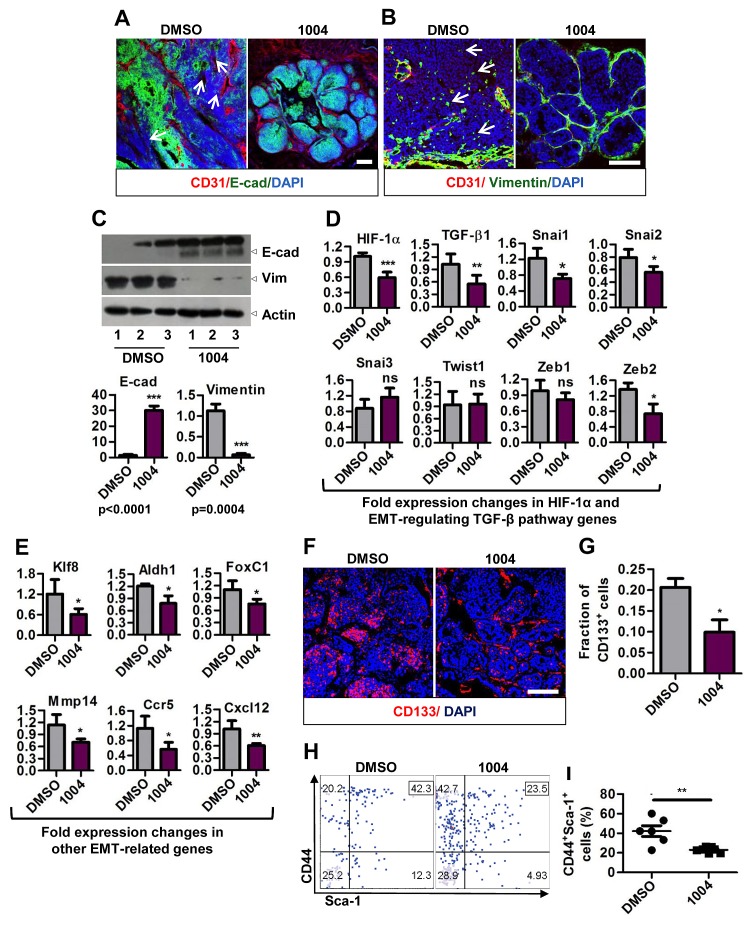
Sac-1004 hinders epithelial-to-mesenchymal transition by affecting related genes and also reduces cancer stem cell population (A) Immunostaining of control or drug-treated MMTV tumor sections (n = 7) for CD31 and E-cadherin. Arrows indicate the patches unstained with E-cadherin. (B) MMTV tumor sections (n = 7) were stained with CD31 and vimentin. Arrows indicate vimentin positive cells in the tumor mass. (C) Western blot of protein samples isolated from control and drug-treated MMTV tumors for E-cadherin and Vimentin (three individual experiments). Quantitation of the blots is shown below. (D and E) Real-time PCR analysis of RNA samples isolated from MMTV tumor (long-term treatment) to show the fold difference in expression of TGF-β pathway (D), and other (E) genes. All gene expressions were normalized to cyclophilin B. (F) Immunostaining of MMTV tumor sections (n = 7) for CD133^+^ cancer stem cells. (G) Quantitation of CD133^+^ cell fractions from the sections stained in (F) using ImageJ software. (H) FACS analysis of tumor cells isolated from MMTV tumors (n = 7; long-term treatment) for CD44^+^Sca-1^+^ cell fraction. (I) Quantitation of CD44^+^Sca-1^+^ cell fraction as shown in (H). Scale bar, 100 μm (in all images). *P<0.05; **P<0.01; ***P<0.001 (Student's t-test). Data are represented as mean ± s.e.m.

Tumor cells fostered under hypoxic and acidotic tumor environment undergo EMT and contribute to metastasis [5, 6]. So we sought to analyze the expression of E-cadherin (epithelial marker) and vimentin (mesenchyme marker) in MMTV tumors. MMTV tumor sections exhibited patchy staining pattern of E-cadherin in control, while Sac-1004 group showed uniform E-cadherin staining (Figure 7A). Also numerous vimentin positive cells were seen in control tumor sections, the number of which decreased sharply in Sac-1004 group (Figure 7B). These findings were also confirmed by Western blotting of tumor proteins (Figure 7C).

Hypoxia and HIF-1α have been shown to play key role in promoting tumor growth and fueling EMT via various pathways (Notch, hedgehog, Wnt, and TGF-β) [5, 6, 32]. Real-time PCR analysis revealed that *Tgf-β1* and its target transcription factors *Snai1, Snai2, and Zeb2* were significantly reduced in Sac-1004 treated MMTV tumors (Figure 7D). Transcription of other genes involved in EMT and metastasis namely *Klf8, Aldh1, FoxC1, Mmp14, Ccr5, and Cxcl12* was also found to be decreased by Sac-1004 treatment (Figure 7E). These results suggest that Sac-1004-mediated leakage inhibition is correlated with reduction in metastasis and this involves downregulation of EMT in tumor cells.

EMT is also responsible for cancer cells acquiring stem cells like properties. These CSCs are critical for promoting tumor growth and contributing to metastasis [5, 32]. Since we observed reduced tumor mass (Figure 5J) and metastasis inhibition upon Sac-1004 treatment, we sought to analyze CSC population in MMTV tumor sections. Immunostaining of MMTV tumor samples for CD133 revealed that nearly 20% of the cell population possessed stem cell character in control tumors; however, the number was reduced to approximately 10% upon Sac-1004 treatment (Figure 7F,G). FACS analysis of tumor cells from MMTV mice (long-term treatment) for CD44^+^Sca1^+^ cell population also confirmed that CSC population was substantially decreased after Sac-1004 treatment (Figure 7H,I). Conclusively, Sac-1004-induced junction potentiation proved to be an efficient way to curb EMT, which might have contributed to the decrease in metastasis and induced-CSCs population of tumor.

## DISCUSSION

Tumor blood vessels are abnormal in structural and functional aspect. Chaotic and non-uniform blood flow give rise to a hostile hypoxic environment where tumor cells bathe in variety of growth promoting factors. Poor perfusion and leaky blood vessels limit the delivery of cytotoxic agents to tumor and thereby decreases chemo/ radio therapeutic efficacy. This results in incomplete therapy and subsequent recurrence of tumor. Further yet, tumor cells that are educated in hypoxic environment are often more viable and resistant to chemo/radio therapy. These cells usually are more aggressive and have a tendency to form metastases [3, 33]. Here, we have demonstrated that direct potentiation of endothelial junction integrity by a small molecule, Sac-1004, is a reliable means to normalize blood vessels, soothe tumor microenvironment, increase the efficacy of cytotoxic agent, and reduce metastasis of tumor cells.

In the recent years, a number of studies have reported the involvement of various factors in tumor vascular normalization [1]. VEGF/VEGFR2 signaling is involved in rapid proliferation of cells and induction of hyperpermeability in blood vessels. Downregulation of this signaling increases tumor oxygenation, increases pericyte coverage, and improves pathologically thickened basement membranes, at least during a time window [8, 34]; Semaphorin3A improves pericyte coverage of blood vessels and restores normoxia [35]; Vasohibin-1 blocks sprouting angiogenesis resulting in mature vessels covered by mural cells [36]. In these cases, normalization seems to be induced by curbing rapid growth of tumor blood vessels. Alternatively, PHD2 haplodeficiency has been shown to affect the endothelial lining and vessel maturation via HIF-driven upregulation of soluble VEGFR-1 and VE-cadherin [37]. It has also been reported that loss of regulator of G-protein signaling 5 (Rgs5) could induce pericyte maturation, vascular normalization, and marked reduction in hypoxia and vessel leakiness [38]. Nonetheless, the relation between vascular integrity and normalization still remains elusive. Interestingly, studies with angiopoietins show that Ang1 could enhance endothelial cell junction integrity at the growing front [8, 10, 13] and inhibition of Ang2 could normalize tumor blood vessels. These reports suggest that junction tightening might be involved in tumor vascular normalization. In the present study, we directly stabilized the tumor endothelial junction by reorganization of actin cytoskeleton with Sac-1004, and this effectively resulted in increased perfusion, reduced hypoxia and vascular normalization. We speculate that hypoxia reduction by Sac-1004 might have affected certain growth factors responsible for the interaction of ECs and pericytes; and this would have caused enhanced pericyte coverage. Hypoxia might also affect the activity of matrix metalloproteases [2] which are responsible for the thickness of basement membrane. Further investigation is required to clarify these issues. Taken together, our study has clearly shown that vascular junction integrity could be a crucial factor for the induction of normalization in tumor blood vessels.

Anomaly in vascular perfusion results in hypoxic tumor microenvironment [2]. Cancer epithelial cells, normally bound to underlying basement membrane, undergo various change in their gene expression resulting in altered adhesive, polarity and migratory properties upon exposure to hypoxia. In the present study, Sac-1004 decreased both distant and regional metastasis in two different mice models: i) MMTV-PyMT and ii) B16BL6 injected mice. This observation is correlated with a previous study that showed inhibition of VE-cadherin-dependent junction disassembly can prevent distant metastasis of cancer cells [39]. Metastasis inhibition by Sac-1004 also coincided with the result that several transcription factors (*Snail, Twist, and Zeb*) associated with EMT were transcriptionally downregulated in Sac-1004-treated tumors. Another component affecting tumor metastasis is cancer stem cell population, which is also responsible for tumor malignancy [40, 41]. We found that Sac-1004 was able to delay the progression of spontaneous breast tumor from hyperplasia to carcinoma stage (Figure 4I). Consistently, a drop in CSC (CD133^+^; CD44^+^Sca1^+^) population was observed in MMTV tumors treated with Sac-1004. We also observed that long-term/late treatment of MMTV mice with Sac-1004 was able to reduce the tumor mass, even though the survival of tumor cells was not found to be affected. We speculate that Sac-1004-mediated decrease in CSC population at transition stage of MMTV tumor (hyperplasia to carcinoma; Figure 7F and H) might contribute to such reducttion [41-43], though further investigation awaits. Altogether, our study suggests that vascular junction potentiation could increase vascular perfusion and reduce hypoxia; which seems to be an efficient way to reduce tumor malignancy by curbing EMT and induced-CSC formation.

The structural feature of tumor blood vessels and tumor microenvironment changes readily with type and stage of tumor. Also various factors, in addition to VEGF, are involved in tumor blood vessel abnormality; the expression of which alters during therapeutic period. This presses an extra degree of difficulty on therapies that target a single factor to control tumor growth. We found that Sac-1004 treatment was able to induce vascular normalization in different tumors types: B16F10 (VEGF sensitive melanoma), LLC (VEGF insensitive lung carcinoma), and MMTV (spontaneous tumor). Also, even when administered at different stage of tumor growth in MMTV mice (8, 10, and 12 week), Sac-1004 was able to reduce vascular hyperpermeability and normalize blood vessels. These phenomena could be correlated with the characteristics of Sac-1004 to affect the common mechanism involved in junction stability, which is converting actin stress fibers to cortical actin ring structure. Thus, unlikely to anti-angiogenic therapies, Sac-1004 could significantly extend the normalization window duration, during which the co-treatment of cytotoxic therapy is known to have additive/ synergistic effect. These results suggest that it might be possible to use Sac-1004, in combination with other therapies, to target various tumor types. Systemic toxicity is another important factor to be considered while developing a new drug. Anti-angiogenic therapies, though highly effective, might also affect normal blood vessels. They are reported to be associated with, in some cases, increased risk of arterial thromboembolic events, hypertension, renal side effects, and impaired wound healing [44, 45]. Considering the endothelial cell protecting and junction stabilizing activity of Sac-1004 [18], it might have relatively less side-effects on normal blood vessels. Indeed, we have not observed any adverse effect on host vasculature, organ functioning, and immune response (CD4, CD8 cell population) in mice receiving Sac-1004 treatment so far (Supplementary Figure S1D-F and Supplementary Table S1). Furthermore, considering the vascular leakage inhibiting activity of Sac-1004 in pathological conditions and its activity against different agonists (VEGF, thrombin, histamine etc.), Sac-1004 could probably be applied for other vascular leakage related diseases such as ischemia/ reperfusion injury, stroke, acute lung injury, acute and chronic inflammation, diabetic retinopathy, macular degeneration etc.

In conclusion, our study establishes that direct inhibition of vascular leakage can be significant to soothe tumor vasculature and limit metastatic spread of tumor cells. This strategy, besides anti-angiogenic therapy, could provide another way to effectively manipulate tumor vessels and microenvironment for therapeutic as well as investigative purpose.

## MATERIALS AND METHODS

### Ethics statement

Investigation has been conducted in accordance with the ethical standards and according to the Declaration of Helsinki and according to national and international guidelines and has been approved by the authors' institutional review board.

### Drugs

Sac-1004 was synthesized as described previously [18]. Working solution of Sac-1004 was prepared by diluting stock (in dimethyl sulfoxide; DMSO) in phosphate buffered saline (PBS). Cisplatin was kindly donated by Prof. Cho Byong Cheol (Severance Cancer Hospital, Korea).

### Pharmacokinetic analysis

Sac-1004 (50 mg/kg) was intravenously injected to eight-week-old male C57BL6 mice. Blood samples were taken from the tail vein at 5 min, 15 min, 30 min, 1, 2, 4, 6, and 8 h. An aliquot of each sample (30 μl) was mixed with verapamil (internal standard) solution (5 μl, 500 ng/ml) and after vortexing briefly, 150 μL of cold methanol was added to each sample. The samples were vortexed again for 1 min and centrifuged for 4 min at 13000 rpm. Supernatant obtained was injected into the HPLC system (80 μl) equipped with an Agilent 6460 triple quadruple MS/MS system and the peak area was recorded.

### Mice

C57BL6 male mice (5/7 weeks old) were purchased from Daehan Biolink (Seoul, Korea) and MMTV-PyMT mice were generously obtained from Prof. Han Woong Lee (Yonsei University, Korea). All the mice were maintained in a laminar airflow cabinet under specific pathogen-free conditions. The facilities were approved by the Association of Assessment and Accreditation of Laboratory Animal Care, and animal experiments were conducted under the institutional guidelines established for the Animal Core Facility at Yonsei University College of Medicine with approval of the institutional care and use committee.

### Diabetic retinopathy model

The mouse model of diabetic retinopathy was generated as previously described [23]. Briefly, 8-week-old C57BL/6 male mice were injected intraperitoneally for 4 consecutive days with 90 mg/kg streptozotocin (Sigma Aldrich, St. Louis, MO) prepared in 0.1M sodium citrate buffer (pH 4.5). Mice were considered diabetic when the blood glucose concentration exceeded 300 mg/dl as measured with a blood glucose test meter (Gluco Dr, All Medicus Co. Ltd., Korea).

To evaluate the effect of Sac-1004 on vessel leakage in diabetic retinopathy, the mice received a 2 μl intravitreal injection of Sac-1004 (1, 5, or 10 μg) in one eye and the vehicle control in the contralateral eye. After 24 h, vascular leakage was quantified by fluorescein angiography. Briefly, 3 mg of 40-kDa FITC-dextran was injected into the left ventricle in a volume of 100 μl and allowed to circulate for 5 min. The eyes were enucleated and fixed in 4% paraformaldehyde for 30 min, and the retinas were dissected out and cut in a Maltese cross configuration. Leakage was viewed under a fluorescence microscope (Carl Zeiss, USA) and quantified.

### Tumor model and treatment regime

B16F10 and LLC cells (5×10^5^ cells/100 μl) were subcutaneously implanted on the lateral flank of 8-week-old C57BL6 mice. B16BL6 cells (3×10^5^ cells/50 μl) were subcutaneously injected in the right footpad of 5-week-old C57BL6 mice. These tumor models and MMTV female mice were injected intravenously with Sac-1004 (50 mg/kg) or an equivalent volume of DMSO (in PBS; 100 μl) as indicated. Mice receiving combination therapy were injected intraperitoneally with cisplatin (2.5 mg/kg) every third day for 2 weeks. Tumor volume was measured with calipers and calculated as width^2^×length×0.523.

### Tumor permeability and vascular perfusion

Tumor permeability was assessed by the Evans blue and the fluorescein isothiocyanate (FITC)-dextran as previously described [19]. Evans blue (50 mg/kg) was injected intravenously and tumors were excised 30 min later. They were dried at 60 ºC for 16 h and then dye was extracted with 1 ml formamide at 55 ºC for 16 h. Absorbance was taken at 620 nm.

Vascular leakage visualization with FITC-dextran was achieved by an intravenous injection of 3 mg/mouse FITC-dextran (40-kDa; Sigma Aldrich) 10 min before capture of tumor. Tumors were then fixed briefly in 4% paraformaldehyde and cryosections were made to observe vascular leakage under fluorescence microscope.

Vessel perfusion was assessed with Hoechst dye (Sigma, 75 mg/kg), injected intravenously 2 min before the mice were sacrificed; and with biotinylated *Lycopersicon esculentum* (tomato) lectin [46], (0.1 mg/mouse; Vector Laboratories) injected 10 min before excision of tumor.

### Histology and immunostaining

To evaluate tumor histology, tumors were excised after perfusing the mice with 1% paraformaldehyde and incubated in 4% paraformaldehyde briefly. After an overnight incubation in 15% and 30% sucrose each, the specimens were embedded in optimal cutting temperature compound and sectioned at 30 μm thickness. Staining was performed as previously described [8] by incubating with one of the following antibodies: goat anti-VE-cadherin (1:100; Santa Cruz), rat anti-CD31 (1:100; BD Pharmingen, Korea), rabbit anti-perilipin (1:100; Abcam), goat anti-ZO-1 (1:100; Santa Cruz), rabbit anti-ColIV (1:100; Chemicon International, Korea), rabbit anti-NG2 (1:500; Millipore, Korea), rabbit anti-desmin (1:100; Abcam), rat anti-F4/80 (1:100; Abcam, Korea), goat anti-Ang1 (1:100; Santa Cruz), rabbit anti-Ang2 (1:100; Santa Cruz), rabbit anti-laminin (1:1000; Thermo Scientific), mouse anti-hypoxia inducible factor (HIF)-1α (1:100; Thermo Scientific), rabbit anti-E-cadherin (1:100; Abcam), rabbit anti-vimentin (1:100; Abcam), rat anti-CD133 (1:500; Millipore), rat anti-LYVE-1 (1:200; eBioscience), mouse anti-cytokeratin-18 (1:100; Abcam), or mouse anti-Ki67 (1:100; BD Pharmingen). The sections were then incubated in Alexa Fluor-conjugated secondary antibodies. Nuclear staining was performed with 4', 6-diamidino-2-phenylindole (DAPI, 1ng/ml), and apoptotic ECs were detected using a terminal deoxynucleotidyl transferase dUTP nick end labeling (TUNEL) kit (Roche, Korea). Sections were then photographed with a confocal microscope (Zeiss LSM 510). Tumor necrosis was determined by hematoxylin and eosin staining.

Hypoxia was detected by pimonidazole adduct formation caused by an intravenous injection of 75 mg/kg pimonidazole (Hypoxyprobe-1, Chemicon) 1 h before capture of tumor. Adducts were stained with a monoclonal antibody directed against pimonidazole (Hypoxyprobe-1 kit, Chemicon).

### Assessment of lung metastasis

Lungs from MMTV mice or B16BL6-injected mice were captured after tracheal perfusion with PBS and Tellyesniczky's fluid (100 ml 32% formalin and 50 ml acetic acid; total volume 1 liter with 70% ethanol) [47] and were placed in Tellyesniczky's fluid for 48 h. Then the lungs were photographed and embedded in optimal cutting temperature compound. Sections of 10 μm thickness separated by a thickness of 100 μm were made to cover whole lung and hematoxylin and eosin staining was performed to assess lung metastasis.

### Cell culture

The human cancer cell lines MCF-7 and HeLa were obtained from Prof. Han-Woong Lee (Yonsei University, Korea). The human cell line A549 and the mouse cancer cell lines B16F10 and LLC were purchased from American Type Cell Culture, B16BL6 cell line was purchased from Korean cell line bank (80006), and human umbilical vein endothelial cells (HUVECs) were isolated from human umbilical cords according to the established protocol [48]. Tumor cells (B16F10, B16BL6, LLC, MCF-7, HeLa) were cultured in Dulbecco's modified Eagle medium (DMEM; Gibco 11995) containing 4.5 g/L glucose, and A549 cells were cultured in RPMI-1640 medium (Gibco 11875). Both media were supple­mented with 10% fetal bovine serum (FBS; HyClone SH30919.03) and 1% penicillin streptomycin (Gibco 15140). HUVECs were cultured in 2% gelatin-coated dishes with endothelial cell basal medium (CC-3156) supplemented with EGM-2 kit (CC-4176; Clonetics, Lonza Walkersville) and 20% FBS; these cells were used through passage 7. All cells were maintained in a 37°C incubator with 5% carbon dioxide.

### Cell proliferation assay

Cell proliferation was determined with the colorimetric 3-(4,5-dimethylthiazol-2-yl)-2,5-diphenyltetrazolium bromide (MTT) assay as previously described [49]. Briefly, B16F10, MCF7, HeLa, and A549 cells were seeded into 24 well plates (2×10^4^ cells/well) in 0.5 ml DMEM containing 10% FBS. The medium was replaced the next day with serum-free DMEM containing Sac-1004 (0.1–40 μg/ml) or the vehicle control (DMSO). After 48 h, 10 μl MTT (5 mg/ml in PBS) was added to the medium, and the cells were incubated for an additional 4 h. Finally, the medium was removed, and the formazan crystals were dissolved in 200 μl DMSO: ethanol (1:1). Absorbance was measured at 570 nm.

### Western blot

Western blot analysis was carried out as previously described [17]. Retina or cell samples were homogenized in RIPA buffer using a sonicator and the lysates were centrifuged at 14,000 rpm for 15 min to collect the supernatant. For tumor specimens, 1 mm^3^ tissue was homogenized in RIPA buffer and centrifuged. Proteins obtained from the supernatants were electrophoresed on 8% sodium dodecyl sulfate-polyacrylamide gels and transferred to nitrocellulose membranes. The primary antibodies used to visualize protein bands were goat anti-VE-cadherin (Santa Cruz Biotechnology), rabbit anti-occludin antibody (Zymed Laboratories Inc., Korea), rabbit anti-E-cadherin (1:1000; Abcam), rabbit anti-vimentin (1:1000; Abcam), and rabbit anti-Ang2 antibody (Santa Cruz Biotechnology). Horseradish peroxidase-conjugated secondary antibodies were obtained from ThermoScientific (Rockford, IL). Protein bands were detected with ECL Western blotting detection kit (Amersham Biosciences, San Francisco, CA) according to the manufacturer's instructions.

### Real time PCR

Total RNA was isolated from MMTV breast tumor samples using RNeasy Plus Universal kit (Qiagen) according to the manufacturer's instructions. cDNA was synthesized and real time PCR was performed on a pikoreal 96 machine (Thermo Scientific) using Maxima SYBR green qPCR master mix (Thermo Scientific). Gene-specific primers were used at a final concentration of 0.5 μM and their sequences are listed in Supplementary Table S2. All qPCR assays were performed in triplicates in at least three independent experiments using 2 different samples.

### FACS

Fluorescence assisted cell sorting (FACS) of mammary tumor cells was performed as described previously [6]. Briefly, mammary gland tumors were harvested from 14-week old MMTV mice and chopped. Tumor tissues were digested with collagenase type 4 (1mg/ml; Worthington) in RPMI 1640 supplemented with 10% FBS and DNase (0.01mg/ml; Sigma) for 20 min at 37°C. The cell suspension was then passed through a 35 μm filter and the flow-through was adjusted to a cell density of 1.5 million cells/ml. Cells were then stained with PE/Cy7-anti-CD44 (IM7; eBiosciences) and PE-anti-Sca-1 (D7; BioLegend) antibodies and subjected to sorting. Live/Dead fixable dead cell Stain kit (Invitrogen) was used to remove the dead cell population. Flow cytometry was performed by FACSCanto II (BD Bioscience) and data was analyzed using Flowjo software (Tree Star).

### Statistical analysis

All statistical analyses were performed using GraphPad Prism (version 5.0; GraphPad Software, La Jolla, CA). Tests for statistical significance were two-sided, and probability values less than 0.05 were considered significant. The Student's *t-*test or ANOVA was used to compare mean values, and results are presented as mean ± SEM or SD.

## SUPPLEMENTARY FIGURES AND TABLES


