# Supplementary material for: Direct endothelial junction restoration results in significant tumor vascular normalization and metastasis inhibition in mice
Source: Oncotarget. 2014 May 2;5(9):2761–77. doi: 10.18632/oncotarget.1942 (PMC4058043; doi:10.18632/oncotarget.1942)
Supplement: Supplementary file 1 [file oncotarget-05-2761-s001.pdf]

Direct endothelial junction restoration results in significant tumor vascular normalization and metastasis inhibition in mice

Supplemental Material

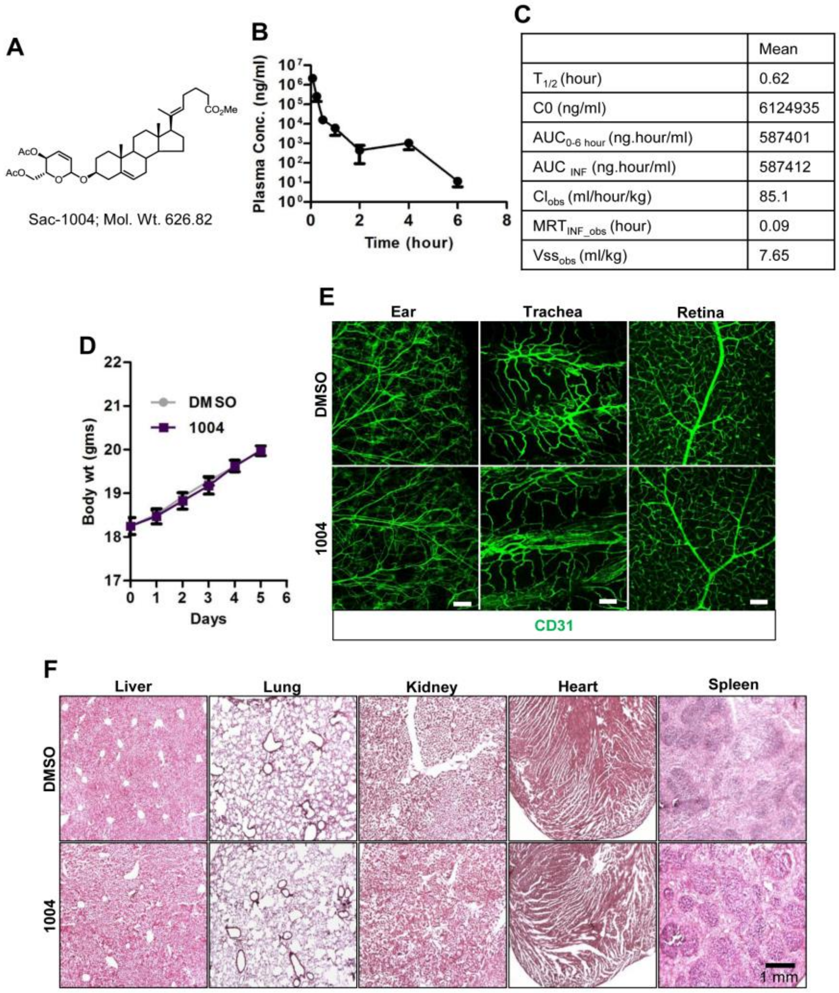

Figure S1, related to Figure 1: Pharmacokinetic and toxicity profile of Sac-1004. (A) Structure of Sac-1004. (B and C) Sac-1004 was intravenously injected into C57BL6 mice (8 weeks; n = 4) and blood samples were taken at indicated time points. Serum level of Sac-1004 was measured by LC-MS/MS. $T_{1/2}$ = Half-Life, C0 = Initial serum concentration, AUC = Area under curve, Cl_{obs} = Observed drug clearance, MRT_{INF_obs} = Observed mean residence time, Vss_{obs} = Apparent volume of distribution at steady state. (D) C57BL6 mice (8 weeks) were intravenously injected with Sac-1004 (1 mg/mice/day) for 5 days continuously and body weight was measured (n = 15 mice per group). (E) Mice (n = 3) were injected with Sac-1004 as in (D) and ear, trachea, and retina were captured 24 h after last injection. Tissues were whole-mount stained with CD31 antibody. Scale bar, 100 µm. (F) Hematoxylin and eosin staining of sections from various organs of mice (n = 3) captured as in (D). Scale bar, 1mm. All data are repeated at least twice.

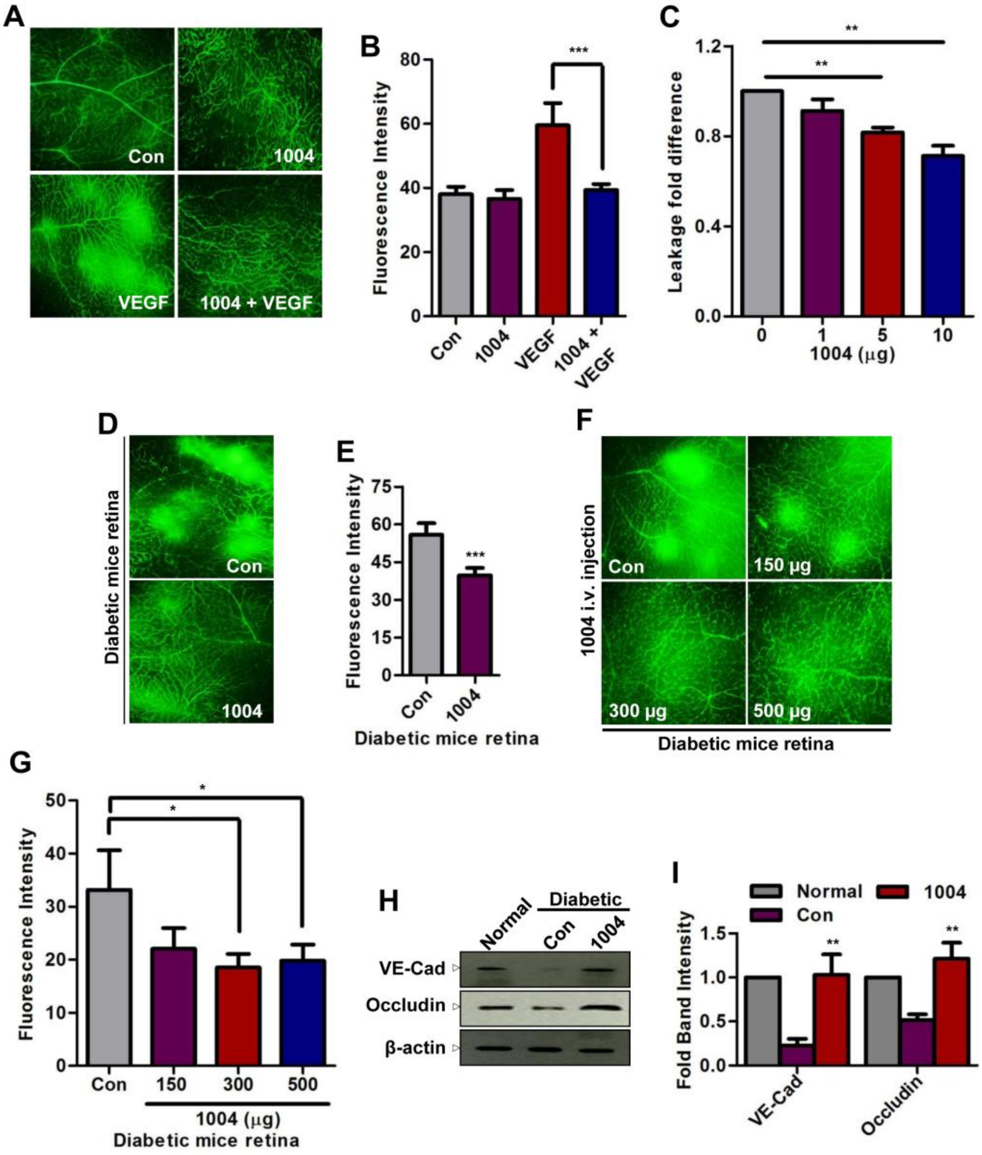

Figure S2, related to Figure 1: Sac-1004 blocks VEGF-induced or diabetic vascular leakage. (A) Adult mouse retina was intravitreally injected with VEGF (50 ng) alone or in combination with Sac-1004 (10 μg). Retinal vascular leakage was assessed by fluorescein angiography as described in the materials and methods (n = 5 mice per group). (B) Retinal vessel leakage in (A) was quantified using ImageJ software. (C) Quantification of vascular leakage in streptozotocin-induced diabetic mouse retina after Sac-1004 treatment. Mice were intravitreally injected with Sac-1004 in one eye, and the vehicle control in the contralateral eye (n = 5 mice per group). (D) Vascular leakage in diabetic mice retinas, intravitreally injected with Sac-1004 (10 μg), was assessed by fluorescein angiography (n = 5 mice per group). (E) Vascular leakage shown in (D) was quantified using ImageJ software. (F) Retinal fluorescein angiography of streptozotocin-induced diabetic mice receiving a single intravenous injection of different doses of Sac-1004 (n = 5 mice per group). (G) Quantitation of vascular leakage shown in (F) using ImageJ software. (H) Western blot analysis of diabetic mouse retinas treated with Sac-1004 or vehicle as described in (D) for VE-cadherin and occludin expression. (I) Quantitation of blots shown in (H) was performed using ImageJ

software. *P<0.05; **P<0.01; ***P<0.001 (Student's *t*-test). All data are repeated at least twice and are represented as mean ± s.e.m.

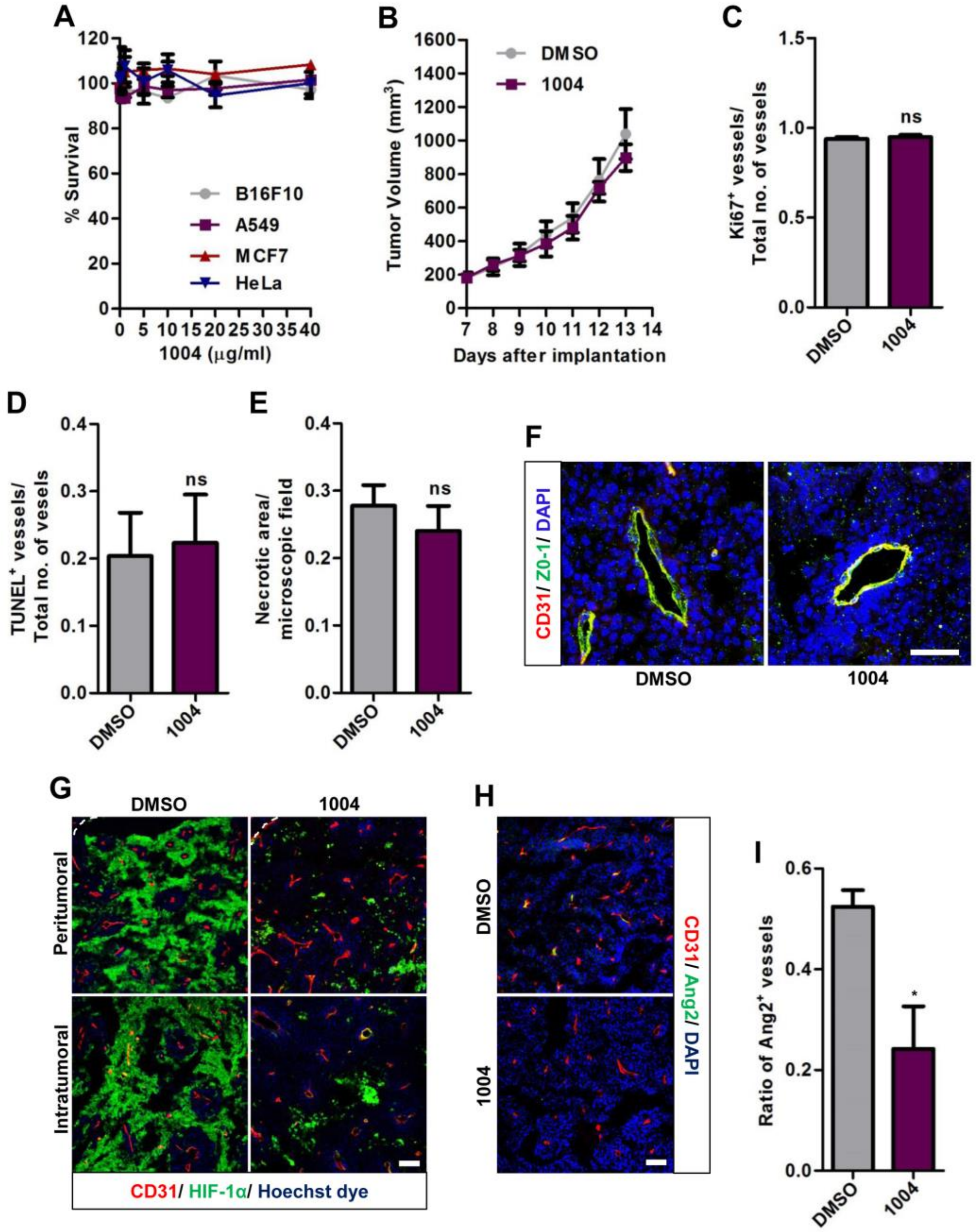

Figure S3, related to Figure 1 and 2: Sac-1004 does not affect tumor cell survival and growth *in vitro* or *in vivo* with no apparent toxicity, while reducing HIF-1α. (A) Survival of various tumor cell lines after Sac-1004 treatment (different concentrations) for 48 h was assessed by MTT assay as described in materials and methods. (B) B16F10 tumor-bearing mice were injected with Sac-1004 (1 mg/mouse) or control (DMSO) and tumor volumes were measured daily (n = 5 mice per group). (C-E) Quantitation of proliferating ECs (C), apoptotic ECs (D), and necrotic area (E) in B16F10 tumor sections treated with two dosages of Sac-1004 or control. Tumor sections were stained against Ki67 (proliferation marker), TUNEL (apoptotic marker), or with hematoxylin and eosin (for necrotic area staining). Random confocal pictures from three sections per tumor (100 μm apart) were quantified using Multi Gauge software. ns, not significant. (F) B16F10 tumor sections, treated with Sac-1004 or

control were immunostained for CD31, ZO-1 and DAPI. Representative images from five individual tumors are shown. Scale bar, 50 µm. (G) Immunostaining of B16F10 tumor sections (n = 5) for CD31 and HIF-1α in the peritumoral and intratumoral region. Hoechst dye indicates perfused vessels. Scale bar, 100 µm. (H) Immunofluorescence staining of B16F10 tumor sections (n = 5) for CD31 and Ang2. Scale bar, 100 µm. (I) Quantitation of the images shown in (H) using ImageJ software. $*P < 0.05$. All data are repeated at least twice and are represented as mean ± s.e.m.

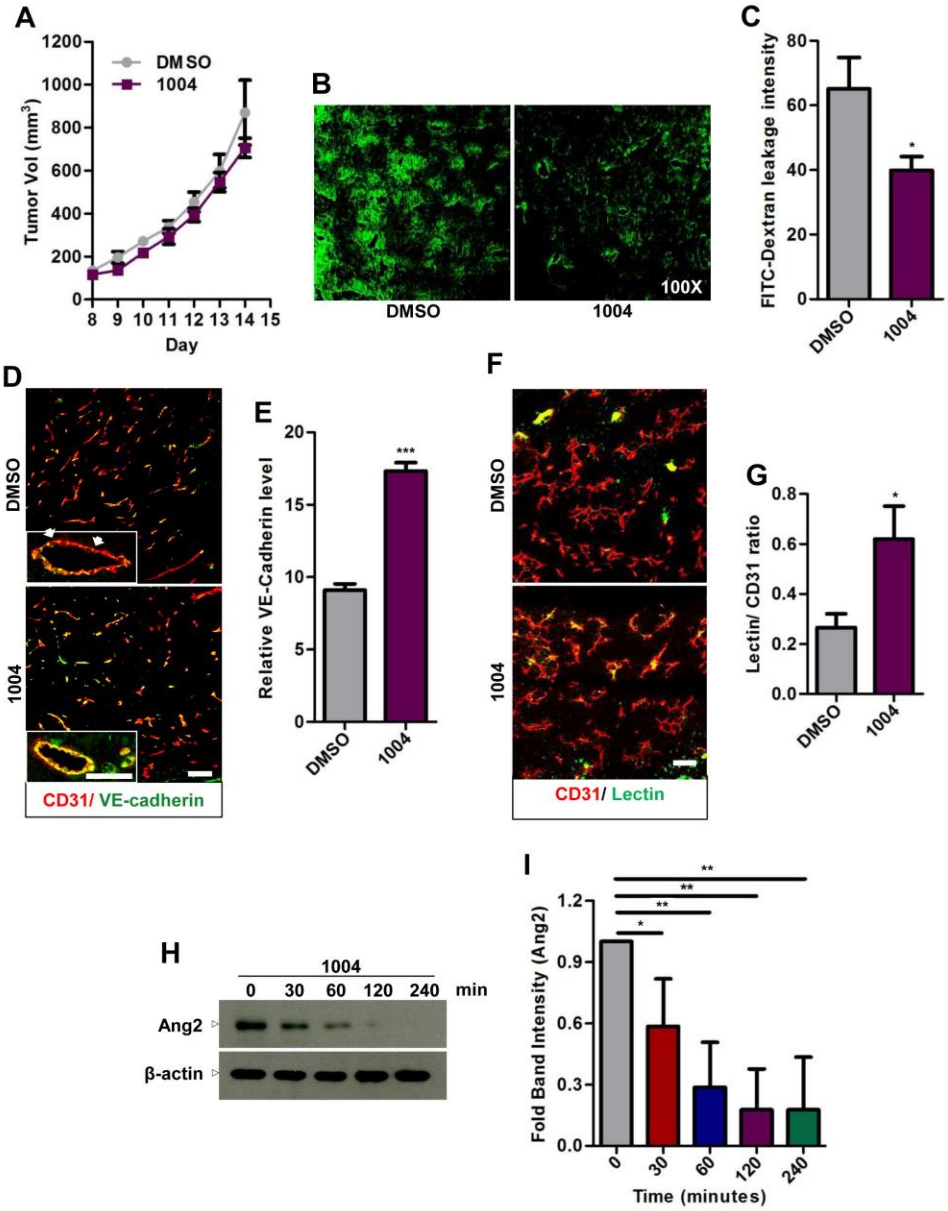

Figure S4, related to Figure 1: Sac-1004 hinders vascular leakage, improves junction integrity, and enhances patency in LLC tumor blood vessels. (A) LLC tumor-bearing mice were injected with Sac-1004 or control (as with B16F10) and tumor volumes were measured daily (n = 5 mice per group). (B) Tumor-bearing mice were injected with Sac-1004 or control and vascular leakage was analyzed by FITC-dextran. Representative images from five individual tumors are shown. (C) Quantification of images shown in (B) using ImageJ software. (D) Immunofluorescence staining of LLC tumor sections (n = 5), treated with Sac-1004 or control as in (A), for CD31 and VE-cadherin. Arrows indicate gaps in VE-cadherin

staining. Scale bar, 100 µm (50 µm in inset). (E) Images shown in (D) were scored using ImageJ software. (F) Immunofluorescence staining of LLC tumor sections, treated with Sac-1004 or control, for CD31 and tomato lectin. Representative images from five different tumors are shown. Scale bar, 100 µm. (G) Images shown in (F) were quantified using ImageJ software. (H) Human umbilical vein endothelial cells (HUVECs) were treated with Sac-1004 (10 µg/ml) and the expression of Ang2 was analyzed at different time points by western blotting. (I) Quantitation of the Ang2 blot shown in (H) using ImageJ software. *P<0.05; **P<0.01; ***P<0.001 (Student's t-test). All data are repeated at least twice and are represented as mean ± s.e.m.

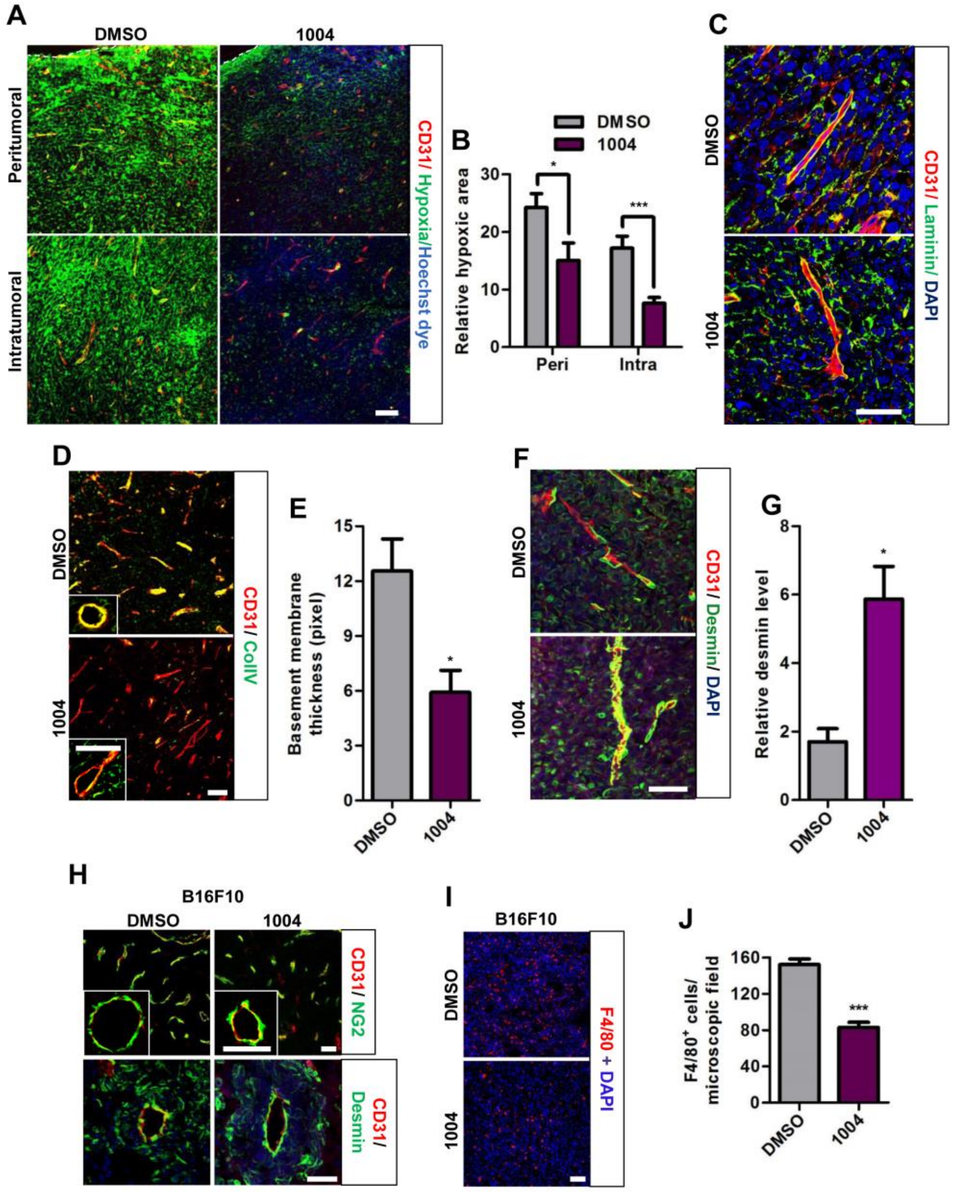

Figure S5, related to Figure 2: Sac-1004 reduces hypoxia and normalizes blood vessels in LLC tumors. (A) LLC tumor sections (n = 5), treated with Sac-1004 or control, were immunostained for CD31, hypoxia and vessel perfusion (Hoechst dye) in peritumoral and intratumoral region. Scale bar, 100 µm. (B) Quantification of hypoxic area as shown in (A) using Multi Gauge software. (C) Immunofluorescence staining of LLC tumor sections (n = 5), treated with Sac-1004 or control, for CD31 and laminin. Scale bar, 50 µm. (D) Immunofluorescence staining of LLC tumor sections (n = 5) for CD31 and ColIV. Scale bar, 100 µm (50 µm in insets). (E) Quantification of basement membrane thickness in LLC tumor

vessels shown in (D) using Multi Gauge software. (F) LLC tumor sections (n = 5) were costained for CD31 and desmin. Scale bar, 50 µm. (G) Level of desmin expression shown in (F) was scored using ImageJ software. (H) Immunofluorescence staining of B16F10 tumor sections (n = 5), treated with Sac-1004 or control, for CD31 and NG2/ desmin. Scale bar, 100 µm (50 µm in insets); Scale bar, 50 µm (bottom figure). (I) Immunostaining of B16F10 tumor sections (n = 5) for F4/80$^+$ macrophages. Scale bar, 100 µm. (J) Quantification of F4/80$^+$ cells in B16F10 tumor sections shown in (I) using ImageJ software. **P*<0.05; ****P*<0.001 (Student's *t*-test). All data are repeated at least twice and are represented as mean ± s.e.m.

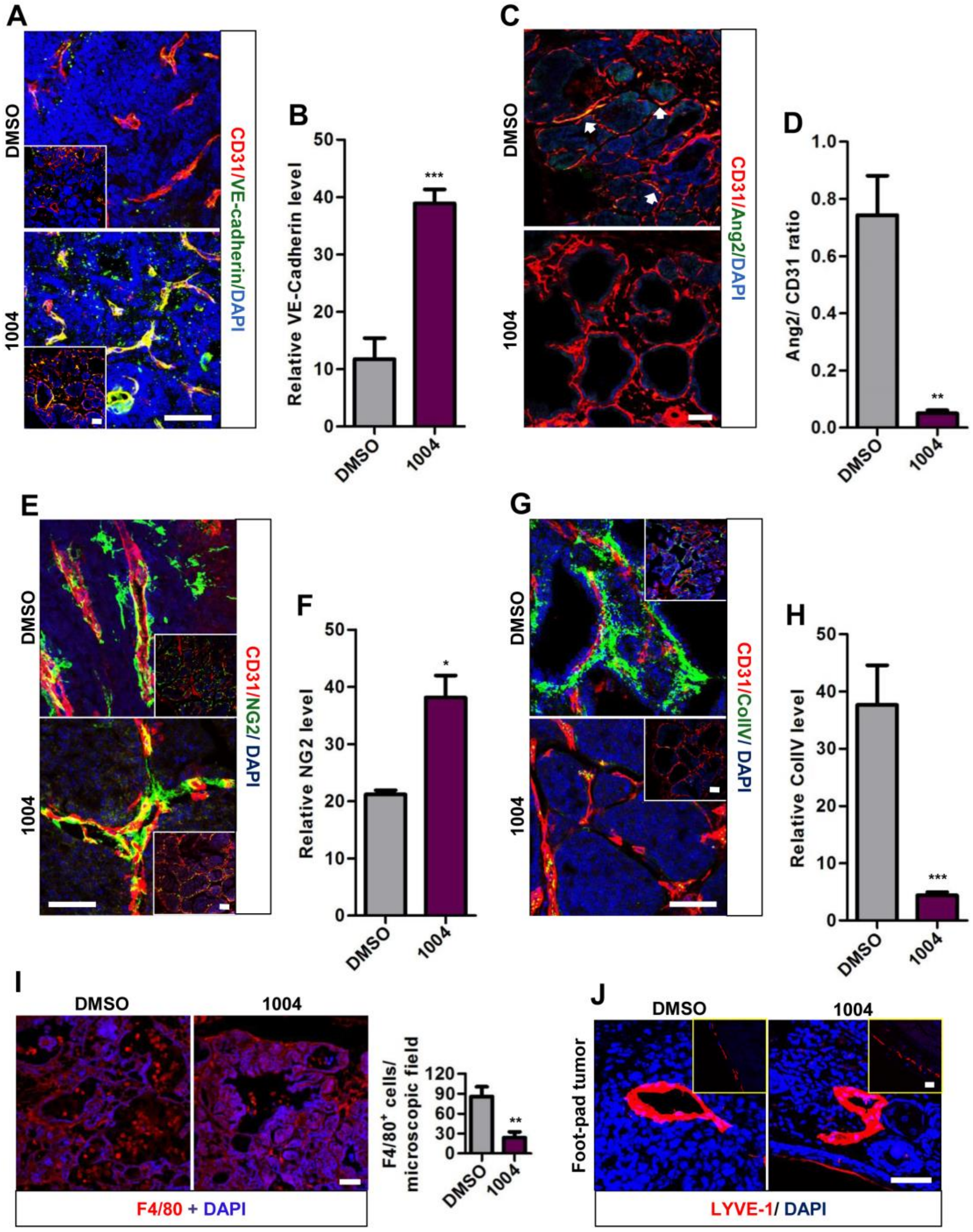

Figure S6, related to Figure 4: Sac-1004 strengthens endothelial cell junction and normalizes blood vessels in MMTV breast tumor. (A) Immunostaining of MMTV tumor section (n = 7 mice) with CD31 and VE-cadherin. Scale bar, 50 µm (100 µm in insets). (B)

Quantitation of relative VE-cadherin level as shown in (A) using ImageJ software. (C) Immunostaining of MMTV tumor sections (n = 7 mice) for CD31 and angiopoietin2. Arrows indicate vessels costained with CD31 and angiopoietin2. Scale bar, 100 µm. (D) Quantitation of the ratio of angiopoietin2 and CD31 positive area as shown in (C) by ImageJ software. (E) Immunostaining of MMTV tumor sections (n = 7 mice) with CD31 and NG2. Scale bar, 50 µm (100 µm in insets). (F) Quantitation of relative pericyte level as shown in (E) using ImageJ software. (G) Immunostaining of MMTV tumor sections (n = 7 mice) with CD31 and ColIV. Scale bar, 50 µm (100 µm in insets). (H) Quantitation of relative ColIV level as shown in (G) using ImageJ software. (I) MMTV tumor sections (n = 7 mice) were stained with F4/80 antibody. Scale bar, 100 µm. They were quantified using ImageJ software. (J) B16BL6 foot pad tumor sections were immunostained for lymphatic vessels. Representative images from 5 different tumors have been shown. Scale bar, 50 µm (100 µm in insets). *$P<0.05$; **$P<0.01$; ***$P<0.001$ (Student's *t*-test). All data are repeated at least twice and are represented as mean ± s.e.m.

Table S1, related to Supplementary Figure S1: Biochemical tests for liver toxicity.

Test	Unit	Normal Range	Cont	Sac-1004 (per mice)		
				1 mg	3 mg	5 mg
ALT	IU/L	28-132	61.54 ± 45.03	40.73 ± 6.35	72.87 ± 46.24	29.21 ± 3.1
AST	U/L	59-247	154.58 ± 78.23	153.8 ± 76.73	182.55 ± 48.01	288.39 ± 22.68
Hb	g/dl	13-18	16.9 ± 1.1	16.8 ± 1.7	16.2 ± 1.3	16.8 ± 1.9

Each group, n = 3. Mice were treated with 5 dosage of control/ Sac-1004 intravenous injections (1dose/ day)

Most parameters in all mice are within normal range except AST at Sac-1004 (5 mg/ mice), which is highlighted by red-color letters.

Normal Range values are derived from the reference in The Laboratory Mouse (2004), edited by H.J., Hedrich and G Bullock, Elsevier Academic Press.

Table S2, related to Figure 7: Real time PCR primers sequences used in this study.

Gene	5'	3'
mHIF-1α	TTTTTGGACACTGGTGGCTCAGCA	CTGTGAATGTGCTGTGATCTGGCA
mTGF-β	CTAATGGTGGACCGCAACAACGC	CGGGCACTGCTTCCCGAATGT
mSnai1	GTCCAGCTGTAACCATGCCT	AAACATCTTTCTCCCGGGGC
mSnai2	CTGTATGGACATCGTCGGCA	ATGGGGGTCTGAAAGCTTGG
mSnai3	CCTTCTCCCGCATGTCTCTC	TGGTGAGCCTCCTGGTGATA
mTwist1	CAGAGATTCCCAGAGGGGCA	GTCAGTGGCTGATTGGCAAG
mZeb1	TCCCGTGCGTTGAGATTTGA	AGTTATGGCTGGGCCAACTC
mZeb2	AGCCCCCGCACATTGTAATA	ATGAGCATGCAAATGCGAGAC
mKlf8	ACTTAACACCAGGCACACAGT	CCAGAGTCAGGACAGATCCC
mAldh1	CTGAGCTCAAGACAGTCGCA	TTGGATAAGAACTGGGGTCACA
mFoxC1	TCCATGAATCAGCACCAGTGT	TGGCATCTGGCTCACAGGTA
mMmp14	CAAGCGACTGCTTTACTGCC	ATGAAGCAATGCTGGGGTGA
mCcr5	AAATCCTACCACACCGGGAC	TTCTGAGGGGCACAACAACT
mCxcl12	TTGCTGTCCAGCTCTGCAGCCTC	ATGTGGCTCTCGAAGAACCGGC
mPpib	TGGGTCCTGTCAATCCCCACACAG	GGGTGTGGTCACCCCATCAGA
